# HRYNet: A Highly Robust YOLO Network for Complex Road Traffic Object Detection

**DOI:** 10.3390/s24020642

**Published:** 2024-01-19

**Authors:** Lindong Tang, Lijun Yun, Zaiqing Chen, Feiyan Cheng

**Affiliations:** 1 College of Information, Yunnan Normal University, Kunming 650500, China; tld1127@163.com (L.T.); zaiqingchen@ynnu.edu.cn (Z.C.); chengfy03@163.com (F.C.); 2 Engineering Research Center of Computer Vision and Intelligent Control Technology, Department of Education, Kumming 650500, China

**Keywords:** autonomous driving, object detection, HRYNet, DFGPN, RMA, LHRYNet

## Abstract

Object detection is a crucial component of the perception system in autonomous driving. However, the road scene presents a highly intricate environment where the visibility and characteristics of traffic targets are susceptible to attenuation and loss due to various complex road scenarios such as lighting conditions, weather conditions, time of day, background elements, and traffic density. Nevertheless, the current object detection network must exhibit more learning capabilities when detecting such targets. This also exacerbates the loss of features during the feature extraction and fusion process, significantly compromising the network’s detection performance on traffic targets. This paper presents a novel methodology by which to overcome the concerns above, namely HRYNet. Firstly, a dual fusion gradual pyramid structure (DFGPN) is introduced, which employs a two-stage gradient fusion strategy to enhance the generation of more comprehensive multi-scale high-level semantic information, strengthen the interconnection between non-adjacent feature layers, and reduce the information gap that exists between them. HRYNet introduces an anti-interference feature extraction module, the residual multi-head self-attention mechanism (RMA). RMA enhances the target information by implementing a characteristic channel weighting policy, thereby reducing background interference and improving the attention capability of the network. Finally, the detection performance of HRYNet was evaluated by utilizing three datasets: the horizontally collected dataset BDD1000K, the UAV high-altitude dataset Visdrone, and a custom dataset. Experimental results demonstrate that HRYNet achieves a higher mAP_0.5 compared with YOLOv8s on the three datasets, with increases of 10.8%, 16.7%, and 5.5%, respectively. To optimize HRYNet for mobile devices, this study presents Lightweight HRYNet (LHRYNet), which effectively reduces the number of model parameters by 2 million. The results demonstrate that LHRYNet outperforms YOLOv8s in terms of mAP_0.5, with improvements of 6.7%, 10.9%, and 2.5% observed on the three datasets, respectively.

## 1. Introduction

The 21st century represents a period characterized by swift and significant progress in science and technology. Autonomous driving [1] embodies a holistic system that amalgamates state-of-the-art technology with the contemporary automotive industry. The development of autonomous driving has considerable significance in the mitigation and prevention of traffic accidents, the resolution of vehicle road safety issues, and the improvement of individuals’ overall well-being. The autonomous driving system has three main components [2]: environmental perception, route decision-making, and motion control. Among these factors, environmental perception is crucial to the overall system, as it directly impacts the efficacy of route decision-making and motion control. The main goal of environmental perception is to recognize and detect target objects effectively. Given the advancements in machine vision algorithms and the development of visible light equipment, an expanding array of companies are employing object detection as the primary approach for perception systems.

Object detection is a crucial task for the perception systems of autonomous driving, aiming to accurately identify and locate traffic objects from images or videos. The detection of traffic objects faces a series of challenges [3]. Firstly, traffic scenes are highly complex, and the influence of different weather conditions, lighting, and traffic situations leads to the attenuation of surface features. Secondly, there is significant scale variation among objects in traffic scenes, requiring algorithms to adapt to different scales. Finally, current object detection networks rely heavily on convolution and pooling operations, exacerbating the loss of feature information between layers. To address these issues, attention mechanisms reinforce the crucial information of targets through assigned weights, enhancing the detection performance of algorithms. Multi-scale processing, by fusing features from different scales, enables algorithms to adapt to targets of various sizes, thereby improving the detection performance of the network.

**Figure 1:**
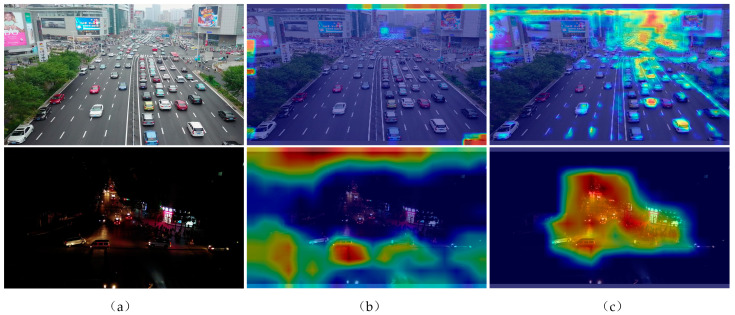
Heatmap comparison. (**a**) A typical real traffic target scene selected from the Visdrone dataset, including dense conditions during the day and fuzzy conditions at night. (**b**) Heat map of the basic algorithm YOLOv8s. (**c**) Heatmap of HRYNet.

Despite the effectiveness of feature fusion strategies and attention mechanisms when enhancing network detection performance, there is still a need for them to adequately address objects with weak feature information. The challenge lies in the diminished characteristic information and the degradation of feature details during the transmission process, leading to suboptimal performance in algorithmic detection, this study introduces the High Robust YOLO network (HRYNet). First, to enhance the network’s anti-interference capability in a highly disruptive environment, HRYNet introduces a novel residual multi-head self-attention mechanism (RMA). The RMA approach mitigates background interference and amplifies target information by employing feature weighting operations. Secondly, the HRYNet introduces a double-fusion gradual pyramid (DFGPN), significantly alleviating the reduction and elimination of target feature information during the feature extraction process, and thereby enhancing the detection performance of the network model in complex background scenarios. The heatmap provides a visual representation of the algorithm’s proficiency when understanding the location and features of the target. Figure 1 displays the actual road scenes captured by the drone and the heatmap comparison between the proposed algorithm HRYNet and the baseline algorithm YOLOv8s. According to Figure 1, it is evident that the proposed HRYNet algorithm in this paper demonstrates enhanced feature learning capabilities, mainly when dealing with traffic targets characterized by limited feature information.

## 2. Related Work

### 2.1. Object Detection Algorithms

Object detection algorithms can be classified into three primary categories according to their underlying principles: those that depend on prior knowledge, machine learning, and deep learning. Perception algorithms that rely on prior knowledge [4] generally consist of two distinct stages: hypothesis generation (HG) and regions of interest (ROI) identification. The HG step generates regions within an image likely to contain the target object. These regions are referred to as ROIs. Hypothesis verification (HV) is a crucial stage in which the algorithm assesses the validity of the regions of interest (ROIs) in terms of whether they contain the target object. While these algorithms frequently demonstrate high accuracy in detecting objects, their effectiveness is diminished in complex traffic scenarios due to their heavy dependence on the intrinsic characteristics of the target objects.

Environmental perception algorithms that utilize machine learning techniques [5] can be deconstructed into three fundamental stages: region selection, feature extraction, and classification. In the first stage, sliding windows of different dimensions select potential regions within the image. The second stage involves extracting pertinent features from the selected regions. Finally, a classifier is utilized in the third stage to identify and classify the extracted features. However, various diverse backgrounds encountered in real-world traffic scenes pose significant challenges when designing a robust classifier. These challenges directly affect the accuracy of classification. Additionally, the computational complexity of this algorithm is high and inefficient, making it unsuitable for traffic scenarios that demand high real-time performance and accuracy. Object detection algorithms, which rely on deep learning techniques, can autonomously acquire and comprehend high-level semantic characteristics of the target object by utilizing convolutional neural networks. Currently, mainstream deep learning object detection algorithms can be classified into two categories: single-stage and two-stage object detection algorithms. Among these, the category of two-stage object detection algorithms is represented by region-based convolutional networks (R-CNN) [6], fast R-CNN [7], and faster R-CNN [8]. These algorithms employ selective search [9] technology to initially identify candidate regions of interest from the image. These regions are then resized before being passed to the CNN for feature extraction. Finally, the system performs classification and regression on the target. Despite the considerable improvements in accuracy and speed compared with conventional algorithms, these two-stage algorithms necessitate multiple iterations of detection and classification, resulting in substantial computational and time demands. Consequently, the two-stage object detection algorithm is unsuitable for object detection in autonomous driving scenarios. In 2015, Joseph Redmon proposed the You Only Look Once (YOLO) [10] network, which introduced the object detection algorithm into a single-stage study. This algorithm partitions the input image into a grid of size S × S, enabling each grid cell to provide information about the bounding box position and the confidence score of the target center within that cell. YOLOv1 demonstrates the potential of single-stage detection, leading to a new era in single-stage object detection. This advancement is primarily exemplified by the Single Shot MultiBox Detector (SSD) [11], the YOLO series [12,13,14,15,16], and RetinaNet [17]. Additionally, the introduction of feature pyramid networks (FPN) [18], attention mechanisms [19,20,21,22,23,24,25], and other structures have further enhanced the detection performance of single-stage object detection algorithms.

### 2.2. Traffic Object Detection Algorithms

There are challenges in autonomous driving object detection, including large-scale variations and complex backgrounds. Numerous scholars have conducted research to address these challenges. Zhou et al. [26] developed a multi-scale target detector incorporating global information aggregation based on coordinates. This approach enhances the network’s ability to detect targets by combining local, global, and coordinate information. Zhang et al. [27] proposed a feature extractor that, through the integration of high- and low-resolution feature maps, generated a comprehensive semantic feature map. Cai et al. [28] introduced a novel feature fusion module that effectively combines the semantic information from the lower layer with the positional information from the upper layer. Shimin Xiong et al. [29] proposed YOLOv3, incorporating spatial pyramid pooling and adaptive spatial feature fusion. This is designed to tackle the notable scale difference between the foreground of autonomous driving and nearby targets, thereby enhancing detection effectiveness. Junfan Wang et al. [30] designed a multi-scale traffic object detector, which assigns weights by information importance and fuses multi-dimensional attention maps to improve feature extraction and information retention capabilities. Yuan Zhang et al. [31]. designed an improved YOLOv7 algorithm, which uses the Res3Unit structure to reorganize the YOLOv7 structure, improve the ability of the network model architecture to obtain more nonlinear features, and solve the problem of the high missed detection rate of vehicle detection. Yingkun Song et al. [32] proposed the MEB–YOLO algorithm, which combines Mosaic and mixed data enhancement, ECA attention mechanism, bidirectional feature pyramid network, and YOLO to solve the problem of the insufficient detection performance of small traffic targets in complex scenes.

### 2.3. Attention Mechanism

The attention mechanism in deep learning is a behavior that emulates the attention characteristics of human vision. It aims to select information most relevant to the current task from a large amount of available information. Numerous exemplary attention mechanisms have emerged with the advancement of research on attention mechanisms. SENet [19], as a representative of the channel attention machine, enhances the network’s feature representation capability by effectively capturing the significant inter-channel relationships. Convolutional block attention modules (CBAM) [20] integrate channel and spatial attention mechanisms to augment the capacity of convolutional neural networks when focusing on significant image feature channels and feature spaces. Coordinate attention (CA) [21] incorporates horizontal and vertical position information into channel attention, enabling the network to attend to various targets selectively and thereby enhancing the network’s capability to learn features effectively. Self-Attention [22] is a technique that improves the attention of a network model by assigning varying weights to global information. Multi-head self-attention (MHSA) [23] utilizes multiple sets of attention weights to perform self-attention transformation on the input feature matrix. This process reduces reliance on external information and enhances internal correlation. The Efficient Multi-Scale Attention Module (EMA) [24] algorithm combines the feature information from two parallel branches by emphasizing dimension interaction. It effectively captures the pairwise relationship between pixels and enhances the pixel-level attention for more advanced feature information. Bi-level routing attention (BRA) [25] modules are used to effectively eliminate predominantly unrelated key-value pairs within broad regions in order to attain enhanced computational allocation and content awareness.

### 2.4. FPN

Multi-scale feature fusion is a crucial strategy in target detection. Combining shallow texture information with deep semantic information enhances its ability to detect targets of varying scales. A widely employed approach for multi-scale feature fusion involves utilizing a feature pyramid network (FPN). FPN [18] facilitates the transmission of semantic information from the upper layer to the lower layer via top-down fusion. However, it does not incorporate the texture information from the lower layer to the upper layer. To address this limitation, PAFPN [33] introduces a bottom-up path to the fusion of deep and shallow information. FPT [34] accomplishes the objective of data enhancement by leveraging the information’s low, medium, and high features, thereby enhancing the connectivity between feature information across different scales. The Graph Feature Pyramid Network (GraphFPN) [35] enables concurrent feature interaction across all scales. However, the fusion technique heavily depends on graph neural networks, significantly increasing model parameters and computational complexity. NASFPN [36] employs neural architecture search techniques to construct an optimal FPN structure. The Asymptotic Feature Pyramid Network (AFPN) [37] is a novel approach that employs a progressive feature pyramid structure. AFPN gradually generates low-, medium-, and high-level features during the top-down feature extraction process in the backbone. This progressive fusion strategy effectively brings information from adjacent feature layers closer together, thereby addressing the issue of a significant gap in non-adjacent layer feature information.

## 3. Methods

### 3.1. Introduction to the Basic Modules

#### 3.1.1. YOLOv8

**Figure 2:**
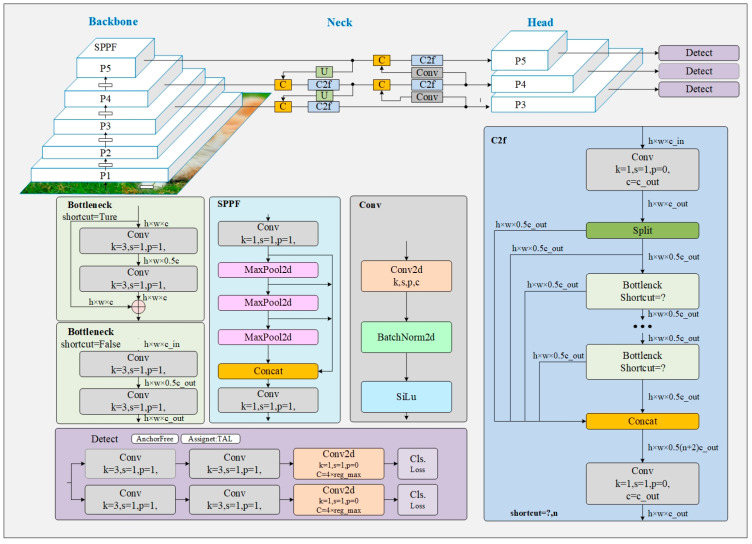
YOLOv8 network structure. ‘?’ indicates whether Shortcut is enabled or not.

YOLOv8 [38] is an enhanced iteration derived from the YOLOv5 version developed by the Ultralytics open-source project, officially launched on 10 January 2023. YOLOv8 demonstrates versatility as a model that effectively addresses detection, classification, and segmentation tasks. The architectural composition is depicted in Figure 2 [38]. It is structured into three main components: the backbone, neck, and head. The backbone component functions as the layer for feature extraction, playing a crucial role in extracting target features of different scales through a top-down pathway. The neck segment is a feature fusion layer, employing the PAFPN strategy to integrate features from various scales. The head section consists of three detect modules designed to detect targets at different scales.

YOLOv8, similar to YOLOv5, can be classified into five models (N, S, M, L, X) with different scaling factors, making them suitable for various application scenarios. It replaces YOLOv5’s C3 structure with a more gradient-flow-rich C2f structure, incorporating the widely adopted decoupled head structure [39] and the anchor-free strategy. Furthermore, YOLOv8 utilizes the task-aligned assigner’s positive and negative sample matching approach, departing from the previous methods of IOU matching or one-sided proportional allocation. In object detection for autonomous driving, the importance of detection performance and speed is equally emphasized. This study utilizes the lightweight YOLOv8s as the fundamental model for conducting experiments to achieve equilibrium.

#### 3.1.2. GSConv

**Figure 3:**
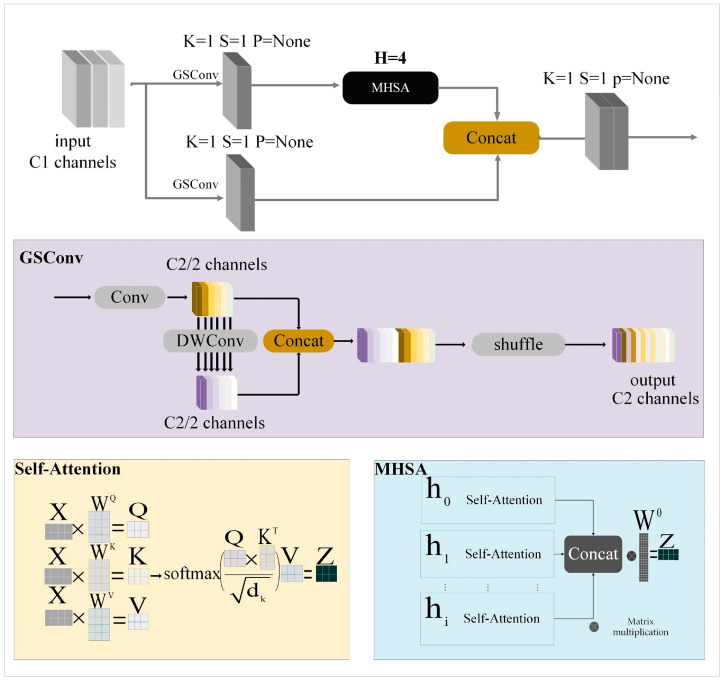
RMA network structure.

GSCONV [40] achieves a harmonious equilibrium between model accuracy and computational efficiency. The purple region in Figure 3 illustrates its composition of deep separable convolution (DSC), standard convolution (SC), and the shuffle operation [41]. The shuffle operation facilitates the exchange of feature information across different channels, thereby effectively integrating the output information of DSC into SC. This approach effectively preserves the inherent advantages of DSC in terms of rapid operation speed.

#### 3.1.3. MHSA

At its core, self-attention involves computing a weighted context vector to represent input sequence information. However, this process tends to overly concentrate attention on internal positions, resulting in a somewhat constrained semantic representation. Multi-head self-attention (MHSA) effectively mitigates this limitation. In contrast to traditional self-attention, MHSA empowers the model to concurrently focus on multiple critical regions, enriching the acquired semantic features. Furthermore, MHSA provides the model with diverse representation subspaces, fostering a more nuanced feature expression, as illustrated in the light blue region in Figure 3. MHSA employs distinct attention weights (W) for linear query, key, and value transformations. These transformed results are parallelly computed using self-attention mechanisms, and the ensuing sets are concatenated. Ultimately, the concatenated results undergo linear transformation with W^0^ to yield the final output.

### 3.2. RMA

The RMA framework is designed to address the problems of gradient vanishing and explosion by providing a structured residual structure. Integrating GSCONV’s exceptional feature extraction capability with MHSA’s outstanding attention capacity leads to a robust detection performance, even in complex scenes. As illustrated in Figure 3, the structure of the RMA consists of two distinct branches. The upper branch first performs GSConv feature extraction, then strengthens the feature layer target information through MHSA. The lower branch performs the GSConv operation only once, to avoid losing target features due to excessive convolution. Finally, the feature maps of the two branches are combined by the concat operation. The GSConvs implemented within the RMA framework exhibit a stride length of 1. This design enhances the module’s capacity to learn local detail features, thereby improving the network’s ability to detect small targets and traffic targets whose feature information is weak. Additionally, it addresses the issue of detection for large-range targets. The MHSA used in this paper has four heads, which provide notable benefits in two critical areas. Firstly, it achieves a harmonious equilibrium between detection accuracy and speed. Secondly, it avoids the issue of inadequate attention capacity resulting from a limited number of self-attention layers and the problem of excessive degradation of feature information caused by an excessive number of layers. In the domain of autonomous driving, where intricate backgrounds introduce diverse interferences to targets, network models require exceptional feature extraction capabilities and feature attention capabilities to address background interference effectively. RMA embodies a successful integration of these two capabilities and has undergone thorough validation through experimental studies. These experiments have showcased its exceptional detection performance in intricate background inspection tasks.

### 3.3. YOLOv8s–FAPN

Undoubtedly, the YOLO series has been successfully applied in various scenarios. In the domain of autonomous driving, feature information traffic targets are frequently obscured due to the intricate nature of the surrounding environment. As the network deepens, there is a risk of losing or weakening the feature information of these targets.

**Figure 4:**
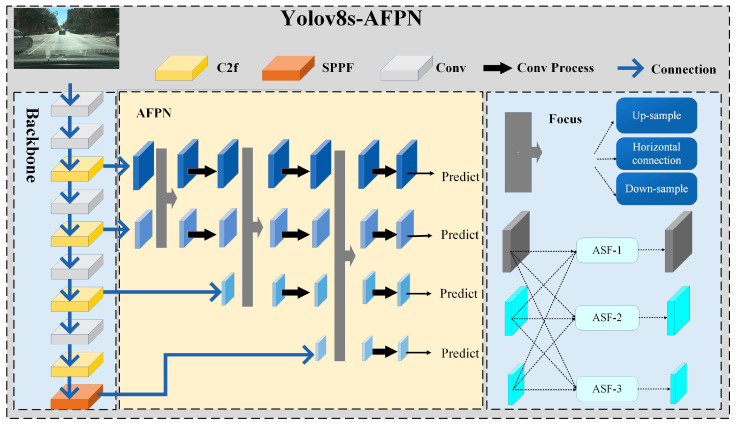
YOLOv8s–AFPN network structure.

YOLOv8s utilizes the Path Aggregation Network (PANet) to integrate shallow texture information with deep semantics, leading to notable enhancements in network performance. However, the top-down feature extraction process and bottom-up feature fusion process of YOLOv8s accelerate the feature attenuation of traffic targets with weak feature information. AFPN, which employs a progressive fusion strategy, aims to tackle the issue of feature attenuation. As illustrated in the yellowish region of Figure 4, the top-down feature extraction process in the backbone involves the progressive generation of low-level, medium-level, and high-level features through AFPN. This process effectively minimizes the disparity in feature information between non-adjacent layers, thereby mitigating the substantial loss of information between these layers.

Consequently, this paper presents a new network architecture called YOLOv8s–AFPN, which combines the backbone region of YOLOv8s with AFPN. This combination is illustrated in Figure 4. The arrow box symbolizes an adaptive spatial fusion operation. The utilization of ASFF, which involves the weighting of feature maps at various levels, guarantees the successful fusion of feature maps across different levels. Experiments have shown that YOLOv8s–AFPN performs better in object detection tasks involving complex road scenarios.

### 3.4. YOLOv8s–DFGPN

**Figure 5:**
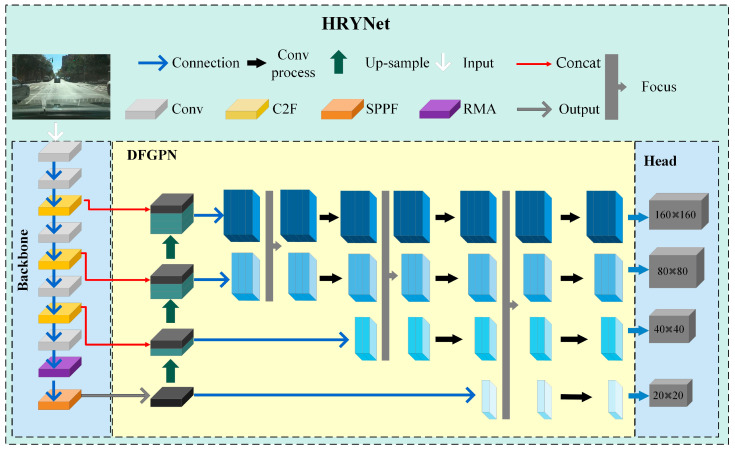
HRYNet network structure.

AFPN addresses the issue of non-adjacent layer feature information loss by the connection of non-adjacent layers. However, this approach also introduces a notable challenge. The recurrent interconnection among various layers imposes constraints on the feature extraction capabilities of individual network layers. In short, AFPN improves the attenuation phenomenon of low, medium, and high feature information by limiting the ability of the feature extraction layer. To tackle this challenge, the present study introduces a pioneering feature fusion strategy known as DFGPN, built upon the foundation of AFPN. The architectural design of the DFGPN is depicted within the delineated yellow region in Figure 5. The underlying principle of DFGPN entails the incorporation of a feature fusion region into the AFPN head. The fusion process involves integrating the foundational semantic information generated by the backbone model with the feature layers at different levels (low, medium, and high) within the backbone region. An integral element of this methodology involves relinquishing the intermediate scale, instead choosing to employ upsampling and fusion techniques to preserve the fundamental semantic characteristics of the final output derived from the backbone. After the concatenation operation, the resulting feature layer incorporates comprehensive information from various target scales and the underlying semantic information, enhancing its effectiveness in target detection tasks. The underlying semantic and shallow features are integrated during the fusion process, generating more robust and multi-scale semantic information. Compared with the AFPN, The DFGPN guarantees the tight integration of each non-adjacent layer and mitigates the information gap between these layers. Additionally, DFGPN addresses the limitation of feature extraction in the gradual process. 

Simultaneously, the final output results of each scale are influenced by the fusion region of the head. These results encompass the semantic information from the low, medium, and high feature layers of the backbone after the gradual process and incorporate the robust semantic information derived from the underlying semantic information output by the backbone after the gradual fusion process. The DFGPN has demonstrated significant advancements in autonomous driving target detection.

### 3.5. HRYNet 

The architecture of HRYNet and its constituent modules is illustrated in Figure 5, delineated into three distinct regions: the feature extraction region (backbone), the DFGPN, and the detection head (head). The backbone region comprises ten feature extraction layers arranged in a top-to-bottom configuration, which include conv, C2f, Sppf, and RMA. Notably, the C2F module is a novel feature extraction component introduced in YOLOv8. It is designed to provide a more lightweight model while offering enhanced gradient information compared with the C3 structure found in YOLOv5. Spatial pyramid pooling (SPPF) enhances the model’s detection capability for objects with varying sizes by pooling and merging feature maps from different receptive fields. This technique is applied to the backbone layer and employs a double fusion strategy, allowing its functions and advantages to be utilized across the entire network. RMA demonstrates exceptional performance in feature learning and attention, as it is strategically positioned on the ninth layer of the backbone for several compelling reasons. Firstly, this placement prevents the degradation of RMA’s attention capacity caused by excessive convolution and pooling operations. Secondly, it maximizes RMA’s influence on the entire network, enhancing its effectiveness. Lastly, the number of parameters in RMA is influenced by the image scale, and locating it in layer nine results in a more lightweight network model. DFGPN utilizes a double-fusion strategy, where the head fusion process gradually combines the final output of the backbone with feature information from layers 6, 4, and 2 sequentially. This approach enhances the network’s ability to detect targets of varying scales and preserves the complete feature information extracted from each feature layer. The head module consists of four detection heads, each designed to detect targets at different resolutions: 4 × 4, 8 × 8, 16 × 16, and 32 × 32. This allows for the detection of a wide range of traffic target sizes in traffic road scenes.

## 4. Experiments

### 4.1. Introduction to Datasets

In order to assess the efficacy of HRYNet in the domain of autonomous driving traffic target detection, this study utilizes the BDD100K dataset [42], an open-source dataset for horizontal acquisition; the Visdrone dataset [43], which consists of high-altitude UAV acquisitions; and a custom dataset to evaluate the model’s performance.

The BDD100K dataset, released by the University of California in 2018, is considered the most comprehensive, diverse, and abundant open-source dataset currently available. Consisting of a comprehensive dataset of 100,000 images, this collection encompasses various temporal variations, weather conditions, and urban backgrounds. As a result, it serves as an optimal choice for the evaluation of the detection performance of HRYNet. Considering computational limitations, this study utilizes a reduced version of the BDD100K dataset, which consists of 10,000 images.

**Figure 6:**
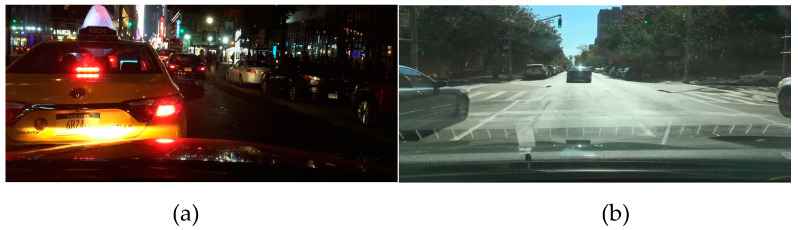
Realistic traffic scenes. (**a**) Traffic target image information in the night scene. (**b**) Traffic target image information in a bright light scene.

The allocation of these images into training, validation, and test sets follows a ratio of 7:2:1. Categories characterized by a limited number of samples are excluded to mitigate potential performance issues with the algorithm caused by inadequate data. The dataset has been categorized into six distinct groups: traffic lights, traffic signs, cars, trucks, people, and riders. Figure 6 presents a visual representation of the image information about traffic targets in intricate road scenarios, effectively demonstrating how the complexity of roads hinders and reduces the distinctive information of these traffic targets.

### 4.2. Evaluation Criteria

In this experimental study, the performance and speed of the network are evaluated using two primary metrics: mean average precision (mAP) and frames per second (FPS). The mAP metric provides an average precision value that encompasses all categories. The computation is derived from the *precision–recall (P–R)* curve, where the x-axis represents recall and the y-axis represents precision. Recall refers to the likelihood of correctly classifying a sample as positive when it is a positive sample. Precision is a measure that indicates the probability of correctly identifying a sample as positive out of all the samples that were predicted as positive. The calculations for precision and recall can be determined using the following formulas:

(1) $$\mathrm{Precision}=\frac{TP}{TP+FP}$$

(2) $$\mathrm{Recall}=\frac{TP}{TP+FN}$$

where *TP* represents how many samples were correctly predicted to be positive, *FP* represents how many samples were incorrectly predicted to be positive, and *FN* represents how many samples were incorrectly predicted to be negative. 

### 4.3. Experimental Environment and Parameter Configuration

The environment used in this experiment was as follows: Windows 10 operating system, RTX3090 graphics card, CUDA (11.6), and Pytorch (1.12). The parameters used in the experiment were as follows: Lr0 = 0.01, Lrf = 0.2, Momentum = 0.937, Batchsize = 16, Epoch = 200.

### 4.4. Methods of Performance Verification

Six sets of experiments were conducted using the BDD100K dataset to assess the effectiveness of each method. The experimental data are displayed in Table 1. The models consist of the baseline algorithms, namely YOLOv3s, YOLOv5s, YOLOv6s, YOLOv7s, and YOLOv8s, as well as the models that have been enhanced by integrating three improved methods, namely AFPN, RMA, and DFGPN, into the networks above. Several observations can be made based on the data presented in the table. Firstly, it is evident that HRYNet exhibits the highest level of detection performance compared with the other models. Compared with YOLOv8s, the proposed model demonstrated a significant performance improvement. Specifically, it achieved a 10.8% increase in mAP_0.5 and a 6% increase in mAP_0.5:0.95. Additionally, in the experiments conducted by group 5, the RMA model exhibited a faster detection speed of 9 f/s compared with YOLOv8s.

Furthermore, through a comprehensive analysis of multiple sets of experimental data, it is evident that incorporating RMA into both the original baseline and improved algorithms consistently enhances the model’s detection performance. This statement highlights the verification of RMA’s generalization ability and emphasizes its practicality in complex backgrounds. Additionally, a comparison between the results of experiments conducted in group 5 and group 6 reveals that HRYNet substantially improves performance indicators, particularly in terms of recall rate (R), when compared with YOLOv8s. The observed increase in recall rate indicates that the implementation of HRYNet significantly improves the capability to identify traffic targets even when provided with limited feature information in complex backgrounds. 

### 4.5. BDD100K Comparative Experiments across Categories

To enhance the credibility of the model’s performance, this paper lists the experimental results of different categories in different models, as illustrated in Table 2. Significant improvements are observed in each category, particularly in the traffic light, traffic sign, and rider categories, which show increases in mAP_0.5 of 14.4%, 13.4%, and 13.2%, respectively. It is worth noting that these three categories have relatively smaller sizes, and that their characteristic information is more prone to loss. This experiment provides further evidence that HRYNet demonstrates exceptional performance when detecting traffic targets with limited and less conspicuous features.

### 4.6. Example of Heatmap Visualization

**Figure 7:**
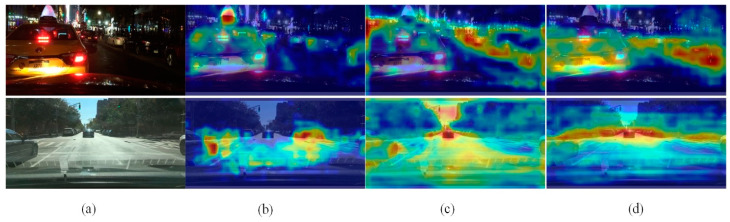
Heat maps for different networks. (**a**) Original image. (**b**) YOLOv8s heat map. (**c**) YOLOv8s–DFGPN heat map. (**d**) HRYNet heat map.

In deep learning object detection, heat maps help to understand which part of an image determine the final classification decision on the output of the model, and they are also a visual display of the performance of the network model detection. The visual representation of the network model’s detection performance is an additional function of the heatmap. To evaluate the feasibility of DFGPN and RMA individually, a selection of representative samples from both daytime and nighttime scenarios were chosen for heat map analysis. As depicted in Figure 7, it is evident that the feature information of the traffic target is significantly obscured in the densely illuminated and strongly lit environment. 

The heat map generated by YOLOv8s exhibits limited range and coverage. Consequently, YOLOv8s demonstrates a diminished capacity for feature extraction and attention when confronted with this particular type of target object. The heat map generated by YOLOv8s–DFGPN shows moderate coverage and moderate divergence. It can be inferred that DFGPN mitigates the issue of feature weakening, thereby enhancing the network model’s ability to extract features from the target. The heat map generated by HRYNet demonstrates the presence of high coverage and concentration, thereby confirming the exceptional feature extraction and attention capabilities of HRYNet. Consequently, HRYNet proves to be more applicable in intricate traffic scenarios.

### 4.7. Comparative Experiment of Detection Effect

**Figure 8:**
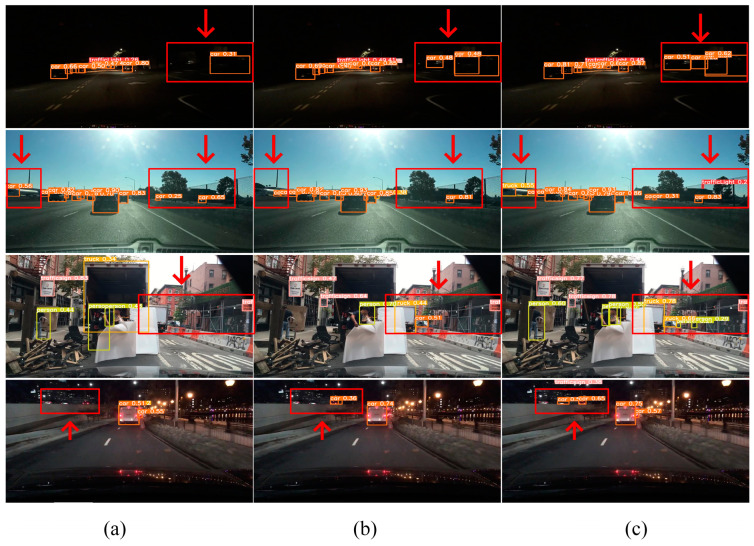
BDD100K actual detection effect on the dataset. The red arrow and border in the figure are post-processing, which aims at a quick and intuitive reading contrast effect. (**a**) Detection effect of the original algorithm YOLOv8s. (**b**) Detection effect of YOLOv8s–DFGPN. (**c**) Detection effect of HRYNet.

To evaluate the feasibility of utilizing DFGPN and RMA in intricate road environments, authentic images captured from diverse, complex roads were chosen to validate the model’s actual detection capabilities, as depicted in Figure 8. A comparison between YOLOv8s and YOLOv8S–DFGPN shows that the latter exhibits greater confidence when detecting identical target objects. Compared with the previous two methods, HRYNet performs better when detecting the same target, exhibiting higher confidence levels. Additionally, HRYNet effectively reduces the missed detection rate and false detection rate. The empirical findings of the detection results provide evidence for the practicality of HRYNet in the automatic driving target detection domain. The proposed method exhibits high robustness when confronted with intricate background interference, making it suitable for various road scenes.

### 4.8. Ablation Experiments

In this study, the control variable method is employed to conduct ablation experiments on the BDD100K datasets. The objective is to analyze the impact of various innovation points on the algorithm’s performance. The results are presented in Table 3, below, with the symbol “√” denoting the utilization of the module optimization method and “×” indicating the absence of module optimization. When comparing experiment 0 and experiment 4, it is evident that the performance indices of the original algorithm YOLOv8 have significantly improved. Specifically, there is a 3% increase in precision (P), an 11% increase in recall (R), a 9.8% increase in mAP_0.5, and a 4.6% increase in mAP_0.5:0.95. These data demonstrate that the utilization of the DFGPN dual fusion strategy effectively enhances the feature aggregation capability of the network model and addresses the issues of the inadequate feature learning ability and feature information attenuation in the original algorithm. Consequently, it significantly enhances the accuracy and recall rate of the network.

When comparing experiments 0, 3, and 5, it becomes evident that the PANet strategy of the original algorithm did not yield satisfactory results for RMA. However, when combined with the DFGPN strategy, the anti-interference capability of RMA was effectively demonstrated across different network areas. As a result, the algorithm’s performance was significantly enhanced, with a 5% increase in P, a 10% increase in R, and a 6% increase in mAP_0.5 and mAP_0.5:0.95. The data analysis indicates that, while YOLOv8s demonstrates satisfactory detection capabilities, its performance is constrained in intricate traffic scenarios, resulting in a significant number of missed and erroneous detections. HRYNet exhibits robust feature extraction capabilities and strong resistance to interference, making it particularly suitable for target detection in autonomous driving.

### 4.9. Experimental Comparison of Improvements in Different Attention Mechanisms

In order to independently validate the suitability of MHSA for autonomous driving target detection in the RMA module, this study replaces MHSA with various attention mechanisms currently considered superior. In order to exclude the interference caused by DFGPN, the module is positioned in the ninth layer of the YOLOv8s–AFPN feature extraction area following replacement. The training outcomes of each residual attention module on the BDD100K dataset are presented in Table 4. Experimental findings demonstrate that using RMA yields superior performance in intricate traffic scenarios. Note: the subscript on the right denotes the origin of this attention mechanism. 

### 4.10. Experimental Results on the VisDrone Dataset 

**Figure 9:**
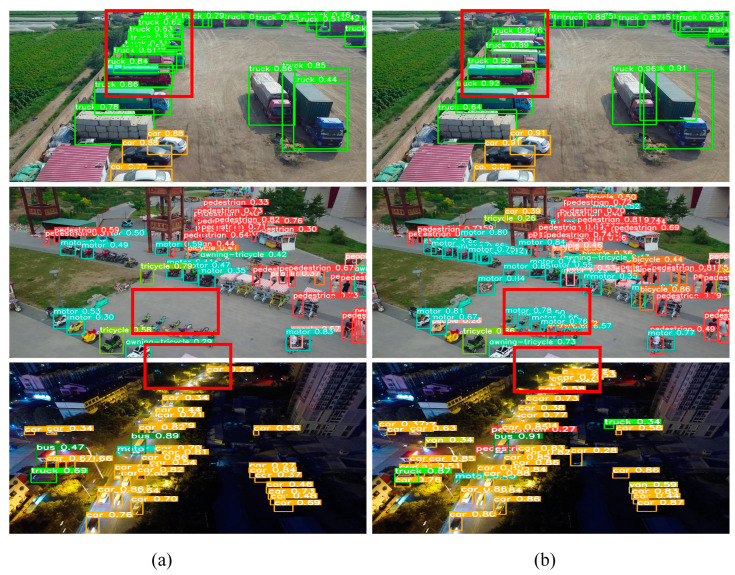
VisDrone actual detection effect on the dataset. The red borders in the figure are post-processing, which aims at a quick and intuitive reading contrast effect. (**a**) Detection effect of the original algorithm YOLOv8s. (**b**) Detection effect of HRYNet.

To enhance the evaluation of the model’s capabilities and minimize the potential influence of dataset bias, this study utilized the VisDrone dataset for conducting tests and training on both YOLOv8s and HRYNet. The VisDrone dataset, acquired by the ALSKYEYE team at Tianjin University through a drone camera, is renowned for its extensive coverage, varied collection environments, and restricted target features. The utilization of HRYNet for assessing the generalization capabilities is an optimal choice. The detection results of both models on the VisDrone dataset are depicted in Figure 9. To facilitate comparison, a red border has been added to the figure. YOLOv8s exhibits significant challenges in terms of both detection and missed detection. In contrast, the HRYNet model demonstrates higher confidence in each target frame, effectively addressing the challenges of detecting small targets and targets with weaker feature information. The experiments confirm that the dual-feature fusion strategy and RMA anti-interference module of HRYNet are more suitable for meeting the requirements of traffic target detection.

### 4.11. Loss Curves Comparison Experiment

**Figure 10:**
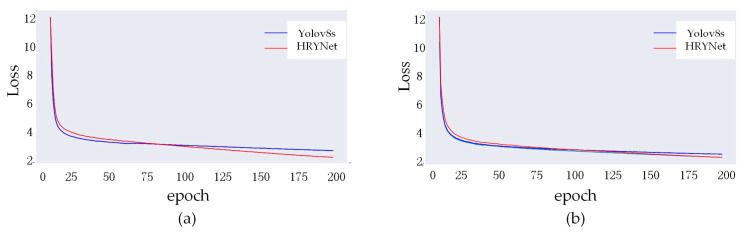
VisDrone actual detection effect on the dataset. (**a**) Loss curve of the BDD100K. (**b**) Loss curve of the VisDrone.

The loss function plays a pivotal role in evaluating the efficacy of a model’s learning process, as it quantifies the discrepancy between the predicted values and the actual values. The loss value in YOLOv8 is calculated as a weighted sum of the classification loss, localization loss, and confidence loss. The loss curves for both algorithms on the two datasets are illustrated in Figure 10, where the x-axis denotes the number of iterations, and the y-axis represents the cumulative loss value. The loss value for both algorithms experienced a substantial decrease during the initial training phases on various datasets. During the training process, it was observed that HRYNet initially had a slightly higher loss compared with YOLOv8s. However, as the training progressed, the loss value for HRYNet gradually surpassed that of YOLOv8s, indicating better performance in the middle and later stages. This observation suggests that HRYNet demonstrates a better learning capability.

### 4.12. Comparative Experiments of Multiple Advanced Models

As the present study utilizes the lightweight BDD100K dataset, comparing the experimental results with the advanced network architectures will not be meaningful. Thus, this paper utilizes the experimental results of HRYNet on the Visdrone dataset to conduct a comparative analysis with other advanced models trained on Visdrone in the same experimental setting. The findings are succinctly presented in the table provided below. According to the findings presented in Table 5, the HRYNet model exhibits superior detection performance compared with the advanced network. Notably, the HRYNet model outperforms the CGMDet network, currently recognized for its exceptional detection performance, by a margin of 4.8 points.

### 4.13. Lightweight Experiments

Increasing the number of channels in a model can result in a substantial increase in parameters and computational complexity. To validate the rationality of the HRYNet structure and eliminate the potential influence of increased parameters on the improvement in detection accuracy, this study adjusted the hyperparameter “width_multiple” (control model width) from 0.50 to 0.15. This adjustment resulted in the creation of the LHRYNet. The findings are presented in Table 6. Compared with the YOLOv8s model, the LHRYNet model demonstrates a reduction of approximately 2 million parameters. Despite this reduction, the LHRYNet model significantly improved mAP_0.5, with an increase of 6.7% and 10.9% on the two datasets, respectively. Even when compared with various algorithm models on the experimental platform of this study, the lightweight HRYNet consistently demonstrates superior detection accuracy while maintaining real-time detection capabilities.

### 4.14. Comparison of Training Results on Custom Datasets

**Figure 11:**
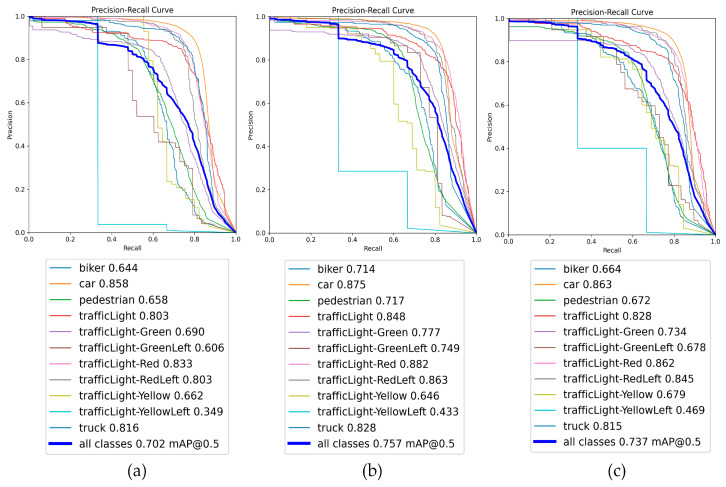
Comparison of experimental results with a customized dataset. (**a**) YOLOv8s experimental results. (**b**) HRYNet experimental results. (**c**) LHRYNet experimental results.

The training results on a custom dataset are further analyzed and validated in this article to assess the detection performance of the proposed algorithm. The custom dataset comprises 8940 images with 11 different categories, providing a diverse and rich collection of images for algorithm performance verification. Figure 11 illustrates the testing results of three algorithms on the custom dataset: YOLO8s, HRYNet, and LHRYNet. The upper part displays the precision–recall (P–R) curves for each category. In contrast, the lower part shows the average precision (AP) values for individual categories and the mean average precision (mAP) across all categories. A comparison between (a) and (b) reveals that HRYNet improves AP_0.5 for various categories, leading to a 5.5% increase in mAP_0.5. Comparing (a) with (c), LHRYNet demonstrates improvements for all categories except for “truck”. The mAP@0.5 has increased by 2.5%. The data presented in this figure indicate a significant enhancement in detection performance compared with YOLOv8s for the proposed algorithm.

### 4.15. Comparison of Detection Effects of Custom Datasets

**Figure 12:**
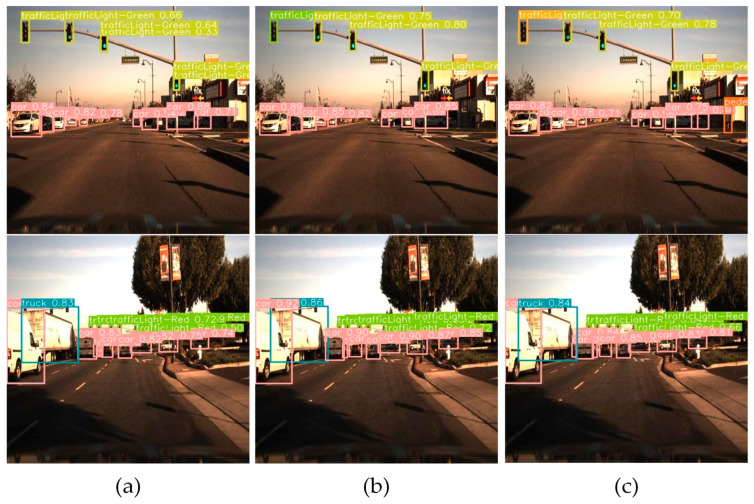
Comparison of detection effects of custom datasets. (**a**) YOLOv8s detection effects. (**b**) HRYNet detection effects. (**c**) LHRYNet detection effects.

Figure 12 illustrates the practical detection performance comparison between the algorithm proposed in this paper and the original baseline algorithm. As shown in the figure, the proposed algorithm demonstrates higher confidence. Furthermore, it highlights the advantages and generalization performance of the proposed algorithm.

## 5. Conclusions

In order to address the problem of the inadequate detection of traffic targets in complex road scenarios, this paper presents HRYNet as a solution. HRYNet introduces the DFGPN as a critical contribution to counter the feature attenuation in complex road scenarios. This novel architecture enhances the network’s capability to extract features from traffic targets, thereby addressing challenges such as detection, missed detection, and false detection. Consequently, it significantly improves the overall performance of detection. RMA was implemented to augment the network’s ability to focus on essential features. This addition contributes to the acquisition of more resilient features and mitigates the impact of intricate road environments, thereby enhancing detection performance. In subsequent research, the primary objective will be to optimize the model to attain an improved equilibrium between detection performance and computational efficiency, thereby enhancing its suitability for implementation in real-world scenarios.

## Figures and Tables

**Table 1:** Performance verification of the methods.

Ordinal	Models	P	R	mAP_0.5	mAP_0.5:0.95	FPS
1	YOLOv3s	0.637	0.451	0.492	0.255	-
	YOLOv3s–AFPN	0.585	0.472	0.505	0.260	-
	YOLOv3s–RMA	0.617	0.473	0.501	0.260	-
	YOLOv3s–AFPN–RMA	0.590	0.470	0.506	0.258	-
2	YOLOv5s	0.630	0.460	0.491	0.244	-
	YOLOv5s–AFPN	0.609	0.506	0.506	0.255	-
	YOLOv5s–RMA	0.649	0.480	0.492	0.246	-
	YOLOv5s–AFPN–RMA	0.268	0.468	0.514	0.265	-
3	YOLOv6s	0.592	0.452	0.477	0.248	-
	YOLOv6s–AFPN	0.608	0.448	0.475	0.245	-
	YOLOv6s–RMA	0.592	0.447	0.476	0.246	-
	YOLOv6s–AFPN–RMA	0.601	0.450	0.475	0.450	-
4	YOLOv7s	0.671	0.493	0.540	0.258	-
	YOLOv7s–RMA	0.633	0.527	0.552	0.266	-
5	YOLOv8s	0.650	0.440	0.496	0.254	60/s
	YOLOv8s–AFPN	0.611	0.480	0.513	0.262	32/s
	YOLOv8s–RMA	0.620	0.460	0.494	0.250	69/s
	YOLOv8s–AFPN–RMA	0.630	0.480	0.529	0.274	32/s
6	YOLOv8s–DFGPN	0.680	0.550	0.594	0.300	19/s
	HRYNet	0.700	0.540	0.604	0.314	20/s

**Table 2:** BDD100K—the detection results for each category.

Models	Traffic Light	Traffic Sign	Car	Truck	Person	Rider	mAP_0.5
YOLOv8s [38]	0.462	0.530	0.739	0.476	0.503	0.267	0.496
HRYNet (ours)	0.606	0.664	0.799	0.554	0.602	0.399	0.604

**Table 3:** Ablation experiments.

Original	AFPN	DFGPN	RMA	P	R	mAP_0.5	mAP_0.5:0.95
0	×	×	×	0.65	0.44	0.496	0.254
1	√	×	×	0.611	0.48	0.513	0.262
2	×	√	×	0.68	0.55	0.594	0.30
3	×	×	√	0.62	0.46	0.494	0.25
4	√	×	√	0.63	0.48	0.529	0.274
5	×	√	√	0.70	0.54	0.604	0.314

**Table 4:** Experimental comparison of different attention mechanisms.

Attention	P	R	mAP_0.5	mAP_0.5:0.95
R-EMA _(cvpr2023)_	0.60	0.473	0.512	0.257
R-TA _(cvpr2020)_	0.62	0.490	0.522	0.269
R-CA _(cvpr2021)_	0.63	0.482	0.523	0.269
R-ECA _(cvpr2020)_	0.635	0.484	0.521	0.269
R-BRA _(cvpr2023)_	0.63	0.485	0.523	0.271
R-SGE _(cvpr2020)_	0.61	0.483	0.521	0.27
R-MHSA (RMA)	0.650	0.487	0.529	0.274

**Table 5:** Model comparison results.

Models	mAP_0.5	mAP_0.5:0.95
RetinaNet [17]	35.9	19.4
Cascade R-CNN [44]	39.9	23.2
Faster R-CNN [8]	40.0	20.6
YOLOv3 [13]	31.4	16.4
YOLOvX [39]	45.0	26.7
YOLOv5l [45]	36.2	20.5
HawkNet [46]	44.3	25.6
Queryder [47]	48.1	28.3
Edge YOLO [48]	44.8	26.2
NWD [49]	40.3	--
ClusDet [50]	50.6	26.7
DMNet [51]	47.6	28.2
CEASC [52]	50.7	28.7
CDMNET [53]	49.5	29.2
YOLOv7 [16]	49.0	28.1
YOLOv8 [38]	39.0	23.0
CGMDet [26]	50.9	29.3
HRYNet (ours)	55.7	35.0

**Table 6:** Experimental results of lightweight models on two datasets.

Dataset	Model	mAP_0.5	mAP_0.5:0.95	Params (M)	FPS (S)
BDD100K	YOLOv8s [38]	0.496	0.254	11.1	60
	HRYNet (ours)	0.604	0.314	80.2	20
	LHRYNet (ours)	0.563	0.29	9.3	33
Visdrone	YOLOv8s [38]	0.390	0.23	11.2	53
	HRYNet [ours]	0.557	0.350	80.2	19
	LHRYNet [ours]	0.499	0.311	9.4	30

## Data Availability

The data used to support the findings of this study are available from the corresponding author upon request.
